# The conformational landscape of transcription intermediates involved in the regulation of the ZMP-sensing riboswitch from *Thermosinus carboxydivorans*

**DOI:** 10.1093/nar/gkaa427

**Published:** 2020-06-01

**Authors:** Oliver Binas, Tatjana Schamber, Harald Schwalbe

**Affiliations:** Institute for Organic Chemistry and Chemical Biology, Center for Biomolecular Magnetic Resonance, Goethe University Frankfurt, Max-von-Laue-Str. 7, 60438 Frankfurt/Main, Germany; Institute for Organic Chemistry and Chemical Biology, Center for Biomolecular Magnetic Resonance, Goethe University Frankfurt, Max-von-Laue-Str. 7, 60438 Frankfurt/Main, Germany; Institute for Organic Chemistry and Chemical Biology, Center for Biomolecular Magnetic Resonance, Goethe University Frankfurt, Max-von-Laue-Str. 7, 60438 Frankfurt/Main, Germany

## Abstract

Recently, prokaryotic riboswitches have been identified that regulate transcription in response to change of the concentration of secondary messengers. The ZMP (5-Aminoimidazole-4-carboxamide ribonucleotide (AICAR))-sensing riboswitch from *Thermosinus carboxydivorans* is a transcriptional ON-switch that is involved in purine and carbon-1 metabolic cycles. Its aptamer domain includes the *pfl* motif, which features a pseudoknot, impeding rho-independent terminator formation upon stabilization by ZMP interaction. We herein investigate the conformational landscape of transcriptional intermediates including the expression platform of this riboswitch and characterize the formation and unfolding of the important pseudoknot structure in the context of increasing length of RNA transcripts. NMR spectroscopic data show that even surprisingly short pre-terminator stems are able to disrupt ligand binding and thus metabolite sensing. We further show that the pseudoknot structure, a prerequisite for ligand binding, is preformed in transcription intermediates up to a certain length. Our results describe the conformational changes of 13 transcription intermediates of increasing length to delineate the change in structure as mRNA is elongated during transcription. We thus determine the length of the key transcription intermediate to which addition of a single nucleotide leads to a drastic drop in ZMP affinity.

## INTRODUCTION

Riboswitches are cis-acting regulatory RNA elements, which sense the concentration variation of metabolites of low molecular weight. They are found in the 5′-untranslated region of mRNA (1), primarily within prokaryotes (2). In general, riboswitches consist of a phylogenetically conserved aptamer domain that undergoes conformational change upon binding a specific ligand, which induces a further allosteric conformational change in a downstream expression platform (3). More than 20 different classes of riboswitches have been identified and have been found to sense metabolites including thiamin pyrophosphate (4,5), adenosylcobalamin (6,7), *S*-adenosylmethionine (8,9) and flavin mononucleotide (10). Typically, ligands sensed by riboswitches are cell metabolites but exceptions including riboswitches sensing fluoride (11) or Mg^2+^ (12) are known. Various riboswitches bind nucleobases such as adenine (13) and guanine (14) or the nucleoside deoxyguanosine (15). Often, riboswitches are found upstream of genes coding for proteins involved in the metabolism or catabolism of the ligand resulting in a feedback loop (16).

Transcriptional riboswitches modulate gene expression during transcription by controlling the formation of rho-independent terminator structures (17). Structural rearrangement of the aptamer domain upon binding leads to formation or destabilization of an antiterminator structure, competing with the terminator structure.

Despite the abundance of crystal structures of metabolite-bound aptamer domains, high-resolution structural information about the full range of transcriptional intermediates and their potentially heterogeneous conformations in the context of co-transcriptional folding are sparse. Switching efficiency between functional ON- and OFF-states in transcriptional riboswitches strongly depends on the time window during which the switch adopts a binding-competent form. In the *Bacillus subtilis* FMN riboswitch, transcriptional pausing is an important mechanism to achieve kinetic control (18). Time-resolved NMR experiments in our laboratory on two different purine-sensing riboswitches, the guanine-sensing riboswitch from *B. subtilis* and the 2′-deoxyguanosine-sensing riboswitch from *Mesoplasma florum* showed that these riboswitches exhibit kinetic control of regulation and the required structural transition to ensure both, functional ON- and OFF-states, are matched to the time window available during transcript elongation (18,19).

The function of second messengers in cells is long-known (20), but their role in riboswitch-based regulation was not identified until the late 2000s when Breaker *et al.* showed that gene expression is regulated by riboswitches that bind cyclic di-GMP (21). Several other conserved second messenger-sensing motifs were identified in the following years, including the *pfl* motif which binds the cellular alarmone ZMP (5-Aminoimidazole-4-carboxamide ribonucleotide (AICAR)) (22).

The ZMP-sensing riboswitch was identified in 2015 and its aptamer fold was named *pfl* motif, as it is frequently associated with a gene coding for pyruvate-formate-lyase (23). As a precursor of inosine, ZMP is involved in the purine and folate biocycle where it is converted into FAICAR by addition of one carbon, provided by 10f-THF. In case of increasing folate stress, ZMP acts as an alarmone, upregulating the expression of associated proteins (24). Regulation is achieved by riboswitches containing the *pfl* motif, which specifically recognize ZMP and ZTP. High ZMP concentrations induce ZMP-binding to the riboswitch, which leads to antiterminator formation and subsequent activation of gene expression. The ZMP-switch is thus considered a transcriptional ON-Switch.

**Figure 1.**
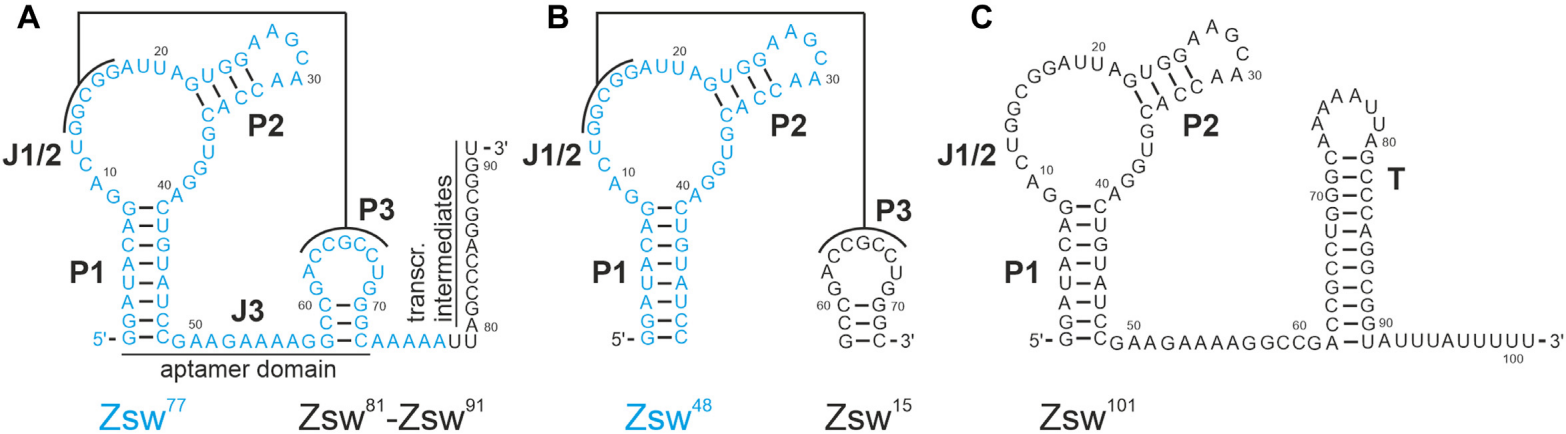
Overview of the secondary structures of the ZMP-sensing riboswitch from *T. carboxydivorans*. (**A**) Secondary structure of the ZMP-sensing riboswitch in pseudoknot form with the construct Zsw^77^ highlighted in blue. Investigated transcriptional intermediates are annotated accordingly. (**B**) Secondary structures of constructs Zsw^48^ and Zsw^15^ used for investigation of pseudoknot formation. (**C**) Secondary structure of Zsw^101^, terminator fold of the ZMP-sensing riboswitch. Stems P1, P2, P3, the Junction J1/2 and the terminator hairpin T are annotated.

The *pfl* motif consists of three helical stems with P2 located in the loop of P1 (Figure 1A). Junction J1/2, located between those helices, forms a pseudoknot with the loop of P3, which features a complementary sequence. Upon ligand binding, the pseudoknot is further stabilized by stacking interactions with ZMP. From the crystal structures it is known that the aromatic ring of ZMP stacks right below the pseudoknot, where it interacts with a U residue of the loop capping P3 (25). Without ZMP, nucleotides within the P3 stem base-pair to downstream residues as transcription proceeds, forming a rho-independent terminator hairpin. The stabilized pseudoknot thus acts as antiterminator, preventing formation of the terminator hairpin (24).

The linker between P1 and P3 is not conserved in length and cannot be resolved in crystal strutures due to structural heterogeneity. Studies by Ferré-d’Amaré *et al.* showed that removal of the linker does not abrogate ZMP binding, though a 10-fold lowered affinity is observed (26).

In a recent study, Lucks *et al.* applied SHAPE-seq (27) to the ZMP-sensing riboswitch from *Clostridium beijerinckii* and described its folding landscape in presence and absence of ZMP (28). They found that riboswitch function is ensured by a labile balance between the conserved short P3 stem, terminator hairpin and pseudoknot stability, which can be strongly perturbed by alteration of a single base-pair in one of those key-players.

In the present study, we characterize the conformations of transcriptional intermediates of the ZMP-sensing riboswitch from *T. carboxydivorans* under equilibrium conditions. These intermediates include the 48 nt containing construct Zsw^48^ encompassing stems P1 and P2, the 77 nt construct Zsw^77^ including stems P1, P2, and P3, ten transcriptional intermediates (Zsw^81^-Zsw^91^, Figure 1A) capable to switch between antiterminator and terminator conformation as well as the full-length riboswitch Zsw^101^. We use NMR spectroscopy as outlined in Fuertig *et al.* (29) to determine the conformations of transcriptional intermediates under varying conditions, employing rapid sample preparation methodology developed by Helmling *et al.* (30) ensuring homogeneity in transcript length. Further, an *in-trans* RNA construct consisting of isolated P1–P2 (Zsw^48^) and P3 (Zsw^15^) was investigated to monitor pseudoknot formation in *trans* (Figure 1B), utilizing differential isotopic labeling for the two RNA constructs for isotope-filtered NMR experiments.

We provide a detailed conformational characterization of the states that are relevant for regulation and that are sufficiently stable to be populated during transcription in order to extend simple two state models describing function of transcriptional ON-switches.

## MATERIALS AND METHODS

### Transcription templates

Transcription templates were generated by PCR from pUC57 vector that contains the full-length native sequence of the ZMP-sensing riboswitch from *T. carboxydivorans* or from previously PCR-amplified DNA. Reverse primers used to obtain templates for Zsw^48^, Zsw^77^ and Zsw^81–91^ are summarized in Table S1 (see Supplementary Material). Primers with high annealing temperatures exceeding 60 °C were chosen for Zsw^101^ as they led to significantly better PCR results. Primers for Zsw^81–91^ contained 2′-methoxy modifications to ensure 3′-end homogeneous transcription products (30). PCR was performed according to the standard protocol by New England Biolabs^®^ (0.5 mM of each primer, 200 mM dNTPs) using home-made Phusion polymerase. As DNA template, we used either 0.1 μl of 1 μM plasmid solution containing the full-length riboswitch sequence or 0.1 μl of PCR-amplified template. Sample integrity was confirmed by native PAGE or agarose gel electrophoresis.

### RNA preparation

All RNAs except Zsw^15^ were prepared by *in vitro* transcription with T7 RNA polymerase (RNAP). PCR-reactions were directly used for transcription without purification. Transcription reactions contained transcription buffer (100 mM Tris/glutamate pH 8.1), 2 mM spermidine, 20 mM dithiothreitol (DTT), 20% (v/v) DMSO, 5 mM of each NTP, 12.5 mM Mg(OAc)_2_, 0.2 u/ml yeast inorganic pyrophosphatase (New England Biolabs^®^) and 144 nM home-made T7 RNAP. Unlabeled NTPs were purchased from Carl Roth GmbH + Co. KG (Germany). ^13^C,^15^N labeled NTPs were purchased from Silantes GmbH (Germany).

The transcripts Zsw^81–91^ were purified according to the protocol developed by Helmling *et al.* (30). After transcription in 10 ml scale, the RNA was washed on 5000 MWCO centrifugal concentrators (Vivaspin 20® from Sartorius AG, Germany). After loading the RNA on the centrifugal concentrator, the first two washing steps were performed with transcription buffer, to remove excess phosphate. Afterwards, the RNA was concentrated to 1 ml and washed at least 10 times with NMR buffer (25 mM potassium phosphate buffer, pH 6.2, 50 mM KCl) in 5 ml steps. Final NMR samples were concentrated to a volume of 250 μl and concentrations ranging from 80 to 120 μM. The RNA was thermally refolded before sample preparation by heating at 60°C for 5 min, subsequent addition of desired amount of magnesium chloride, heating at 40°C for 5min and cooling on ice for 30 min.

The RNA constructs Zsw^77^ and Zsw^48^ were purified by denaturing PAGE. The transcription mixture was loaded on a 3000 MWCO centrifugal concentrator (Vivaspin 20® from Sartorius AG, Germany) and washed repeatedly with water until the elute did not show a significant signal at 180–220 nm in the UV-VIS spectrum (NanoDrop One, ThermoFischer Scientific). The solution was concentrated to 1 ml, mixed with 30 % glycerol and loaded onto a 15% polyacrylamide (PAA) gel (7 M urea). The RNA band was excised from the gel and eluted in 0.6 M NaOAc, pH 5.5. The RNA was precipitated once with cold EtOH and twice with 2 % (w/v) LiClO_4_ in acetone. After resuspending in water, buffer exchange to NMR buffer (as described above) was performed on a centrifugal concentrator.

Zsw^15^ RNA was bought from Dharmacon GmbH. The RNA was deprotected according to the supplier protocol. Following HPLC purification, the HPLC buffer was removed on a 1000 MWCO centrifugal concentrator. After one EtOH and two LiClO_4_ precipitations (see above), the RNA was resuspended in water and the buffer exchanged to NMR buffer. The NMR buffer contained 25 mM potassium phosphate buffer adjusted to a pH of 6.2 and 50 mM KCl. The RNA was thermally refolded before sample preparation.

### NMR spectroscopy

NMR samples were prepared by adding 10 % D_2_O and 7.5 nmol DSS as internal reference to RNA stock solutions in NMR buffer (see above). All spectra were recorded of 280 μl samples in Shigemi NMR tubes (Shigemi Inc.).

NMR experiments were conducted on Bruker AV600, AV700 and AV800 spectrometers, equipped with cryogenic probes. Data were processed with Bruker Topspin 3.5 (Bruker Biospin) and sparky 3.14 (31). Water suppression was achieved using WATERGATE (32) or jump-and-return echo (33) water suppression pulse schemes. ^15^N-editing in ^1^H,^1^H-NOESY was achieved by implementing X-filter schemes before and after t_1_ chemical shift evolution (34). Analysis of dissociation constants and binding competent fraction were carried out by measuring intensities of the imino proton signal of U42 in ^1^H-1D spectra, normalization of values and subsequent fitting while leaving the RNA concentration variable.

### ITC measurements

RNA samples for ITC measurements were essentially prepared as for NMR spectroscopy. ITC measurements were performed on a Malvern^®^ MicroCal iTC200 instrument with RNA sample concentrations of 60 μM and ligand concentrations of 600 μM (for Zsw^77^ and Zsw^91^) or 300 μM (for constructs Zsw^81–83^). Raw data were exported and analyzed using NITPIC (35) and SEDPHAT (36). Fractions bound were obtained from leaving the n-value variable in the fitting procedure.

## RESULTS

### NMR resonance assignment

**Figure 2.**
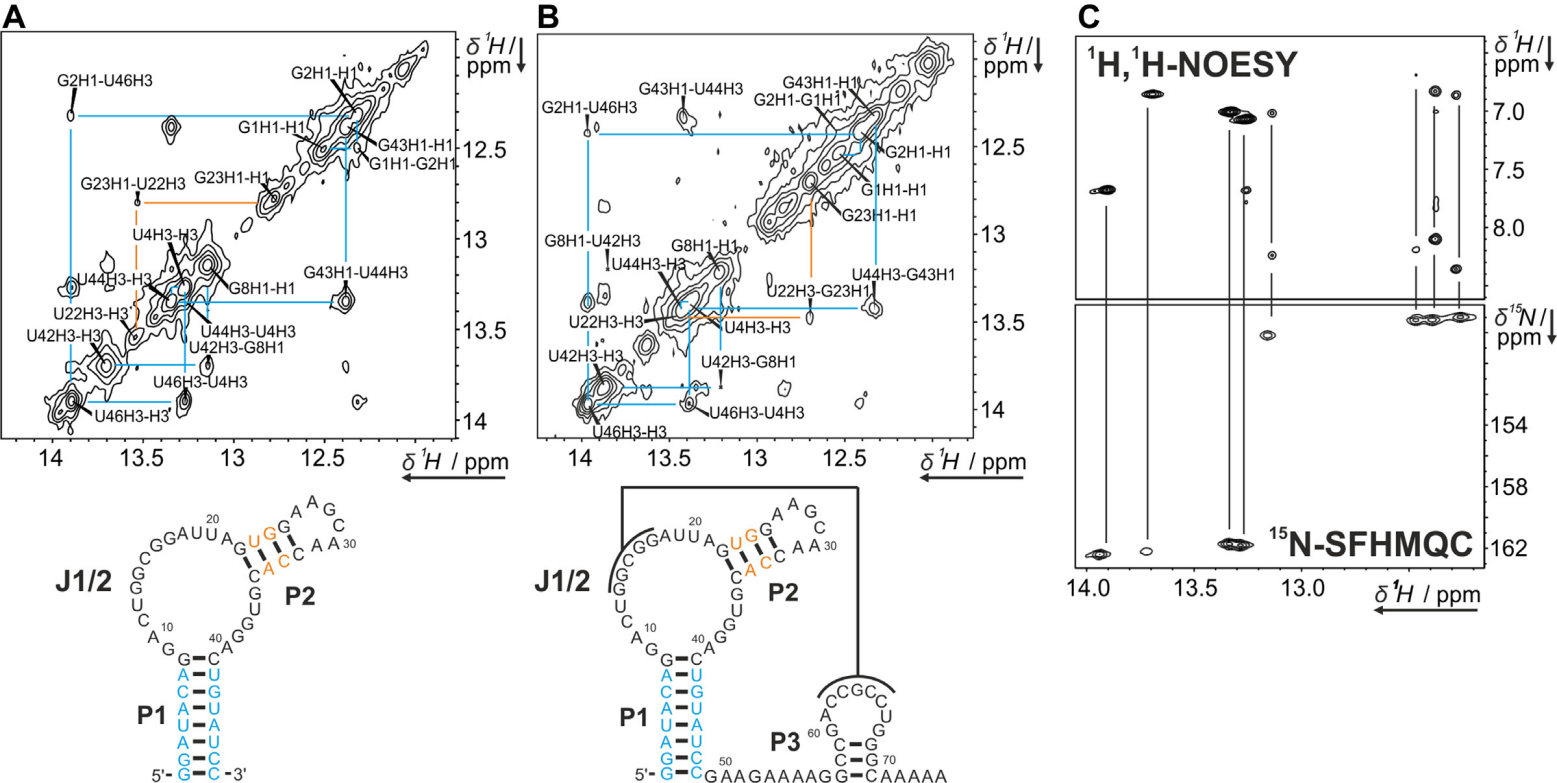
Imino regions of ^1^H,^1^H-NOESY spectra of Zsw^48^ (**A**) and Zsw^77^ (**B**) annotated with assignment. Color-coding indicates resonances from either P1 (cyan) or P2 (orange). Data were measured at 600 MHz, 2048 × 640 points and 184 scans and 800 MHz, 2048 × 512 and 256 points for Zsw^48^ and Zsw^77^, respectively. Samples contained 800 μM (Zsw^48^) or 400 μM (Zsw^77^) RNA, 25 mM potassium phosphate buffer (pH 6.2), 50 mM KCl and 10 % D_2_O. Larger representations of the spectra are shown in Supplementary Figure S1 (**C**) Imino to aromatic region of the ^1^H,^1^H-NOESY of Zsw^48^ (see A) and ^15^N-SOFAST-HMQC (^15^N-SFHMQC) of Zsw^48^, showing ^15^N chemical shift differences for G and U residues. ^15^N-SOFAST-HMQC was measured at 700 MHz, room temperature, 2048 × 128 points and 16 scans on a sample containing 400 μM ^13^C, ^15^N-labeled RNA in 25 mM potassium phosphate buffer (pH 6.2), 50 mM KCl and 10 % D_2_O. Subtle differences in chemical shift are a result of slight temperature differences between spectrometers.

NMR signals of longer RNA constructs can often be dissected to arise from structural elements already present in shorter constructs following a divide-and-conquer-strategy (29). Since stems P1 and P2 are present in Zsw^48^ and in Zsw^77^ (see Figure 1A and b), comparable sets of imino signals are expected for these constructs and could in fact be observed in ^1^H-1D as well as ^1^H,^1^H-2D NOESY experiments (Figure 2). By contrast, NMR signals at 10.5, 11.6 and 12.8 ppm, observable in Zsw^15^, were also observed in Zsw^77^ but not in Zsw^48^.

For ^1^H proton chemical shift assignment, ^1^H,^1^H-NOESY experiments were conducted mapping out the interaction of imino protons with adjacent protons less than 5 Å apart from each other and led to the sequential assignment of G and U residues in P1 and P2 stems. Distinction between signals arising from G and U residues, respectively, was achieved by analysis of the cross-signals to the opposing base, which are two C amino protons for G-C base-pairs, but only one aromatic adenine H2 proton for A-U base pairs; and confirmed by ^1^H,^15^N-correlation spectra on an isotopically labeled sample of Zsw^48^ (Figure 2C). Imino protons of the terminal helical base-pairs in P1 and P2 could not be observed due to solvent exchange. Since the P2 helix is not involved in ligand interaction or pseudoknot formation, we omitted further characterization of this stem. For helix P3 in Zsw^77^ and Zsw^15^ (Figure 1B), no sequential imino proton NOEs were observed, most likely due to the rapid solvent exchange in this very short stem. Subsequently, the assignment of Zsw^48^ was transferred to Zsw^77^ (Figure 2A and B). In summary, we established the conformations for both Zsw^77^ and Zsw^48^. Further imino proton signals were observed at 12.1 and 12.7 ppm for Zsw^48^ and an additional signal at 12.3 ppm for Zsw^77^. These signals likely arise from residues located in the non-helical regions between P1 and P2, since they show no NOESY cross peaks to other imino proton signals.

### Investigation of pseudoknot interaction via *in-trans* construct

**Figure 3.**
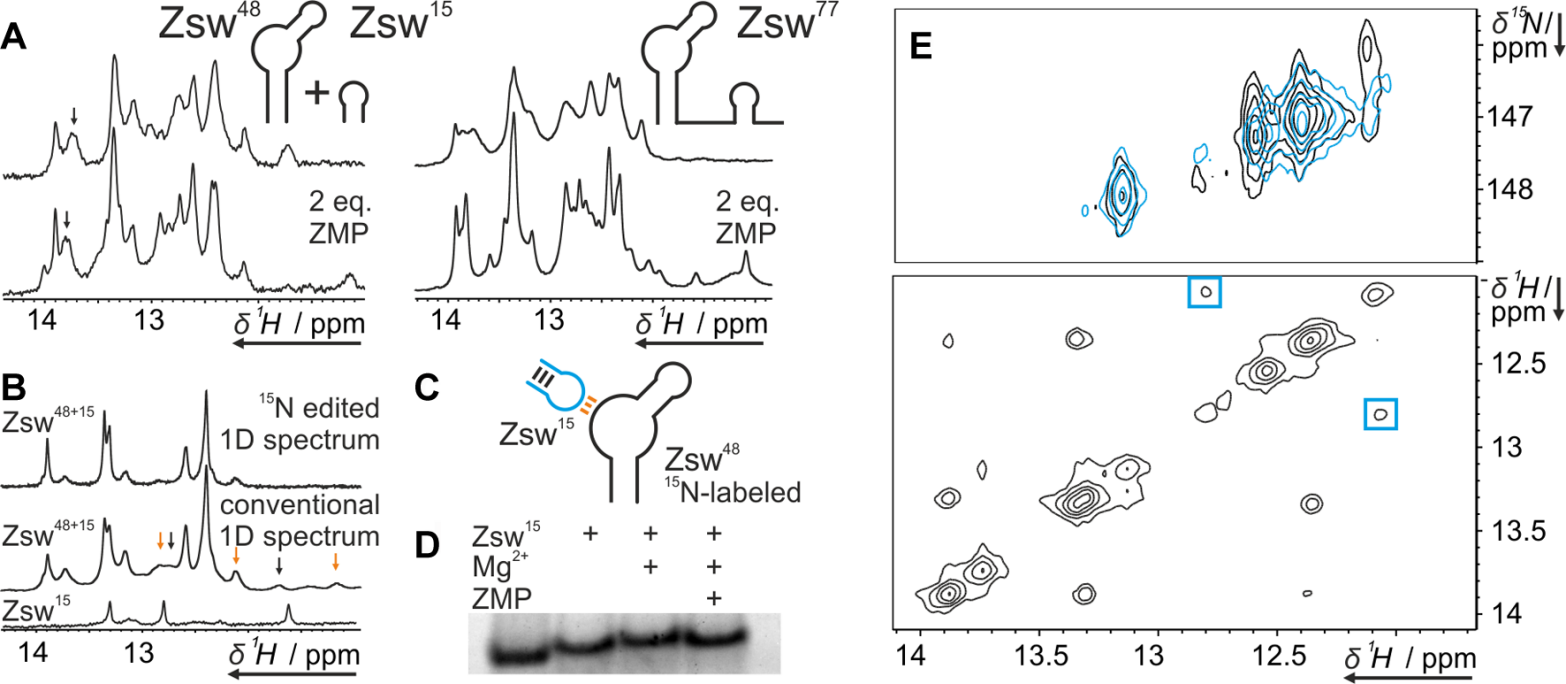
(**A**) ^1^H-1D NMR spectra of the *in-trans* construct Zsw^48^+Zsw^15^ and Zsw^77^ before and after addition of 2 eq. ZMP in presence of 10 mM Mg^2+^. (**B**) ^15^N-edited and conventional ^1^H-1D NMR spectra of ^15^N-Zsw^48^+^14^N-Zsw^15^ and conventional ^1^H-1D of Zsw^15^ in the presence of 10 mM Mg^2+^. Peaks that arise only in the conventional spectrum marked with arrows. Samples contained 100 μM RNA. Stacked and enlarged representations of those spectra are shown in Supplementary Figure S2. (**C**) Schematic representation of the labelling scheme of ^15^N-Zsw^48^+^14^N-Zsw^15^. Colors of base-pairs according to arrow colors in (**B**). (**D**) 12 % native PAGE of Zsw^48^ in the presence of 1eq Zsw^15^, 10 mM Mg^2+^ and 1 eq. ZMP. Pockets were loaded with 200 nmol Zsw^48^ and additions as indicated. The gel is displayed enlarged in Supplementary Figure S3 (**E**) Top: ^15^N-SOFAST-HMQC spectrum (37) of Zsw^48^ before (cyan) and after (black) addition of Zsw^15^ in the presence of 10 mM Mg^2+^. Spectra were recorded at 800 MHz at room temperature with 2048 × 128 points and 8 scans. Bottom: ^15^N-edited ^1^H,^1^H-NOESY of Zsw^48^+Zsw^15^ with the cross peaks that arise from addition of Zsw^15^ marked blue. Spectra were recorded at 800 MHz with 2048 × 432 points and 256 scans.

To investigate the formation of the pseudoknot between J1/2 and P3, which is the essential interaction stabilizing the antiterminator conformation, we designed two RNA constructs Zsw^48^ and Zsw^15^. Zsw^48^ consisted of the P1 and P2 stem including the junction J1/2, and Zsw^15^ consisted of the P3 helix with the corresponding loop. To assess whether these two RNA strands reconstruct the aptamer domain *in-trans*, we added one equivalent of Zsw^15^ to Zsw^48^ and monitored chemical shifts of imino proton signals in the presence of 10 mM Mg^2+^ and 2 eq. ZMP (Figure 3A). Imino proton spectra of the *in-trans* construct Zsw^48^+Zsw^15^ showed no difference to spectra of Zsw^77^, confirming that the pseudoknot formation does not require the linker J3. However, spectra also showed that ZMP affinity is higher for the *in-cis* construct Zsw^77^ than for the *in-trans* construct Zsw^48^+Zsw^15^. The linewidth of reporter signal U42 was larger for Zsw^48^+Zsw^15^, indicating conformational heterogeneity involving a residual unbound conformation, while U42 in Zsw^77^ showed a significant chemical shift perturbation (CSP) of 0.06 ppm. Strikingly, however, Zsw^48^+Zsw^15^ showed clear ligand binding (Figure 3A).

We prepared ^13^C,^15^N-isotope labeled Zsw^48^ and added unlabeled Zsw^15^ to further characterize the pseudoknot interaction. We recorded ^15^N-edited ^1^H-1D spectra as well as conventional ^1^H-1D spectra of all constructs (Figure 3B upper and middle spectra, respectively). In ^15^N-edited spectra, peaks of only those ^1^H can be observed that are covalently bound to ^15^N, which includes only signals of residues in Zsw^48^. In the ^1^H-1D spectrum, several signals were observed which could not be attributed to Zsw^48^ since they did appear only in conventional ^1^H-1D-, but not in ^15^N-edited spectra. Of these, signals at 11.7 ppm and 12.7 ppm (Figure 3B, black arrows) were assigned to the stem imino protons of Zsw^15^, as they closely resemble the signals observed in pure Zsw^15^ (Figure 3B lower spectrum). Signals at 12 ppm and 12.8 ppm belonged to Watson-Crick type base-pairs between Zsw^48^ and Zsw^15^ indicative of pseudoknot formation (Figure 3B, orange arrows). Following up on this observation, we compared ^1^H,^15^N-correlation spectra before and after addition of Zsw^15^ and observed an additional imino resonance at 12 ppm obscured by another ^1^H resonance, which had not been detected in 1D experiments and only appears upon addition of Zsw^15^ and therefor pseudoknot formation. A cross peak from this resonance to 12.8 ppm was observed in the ^15^N-edited ^1^H,^1^H-NOESY of ^15^N-Zsw^48^+^14^N-Zsw^15^ (Figure 3E) proving the formation of two consecutive Watson-crick type G-C base-pairs of the pseudoknot.

These NMR results were supported by native PAGE. By native PAGE (Figure 3D), we detected a slower migration speed for Zsw^48^+Zsw^15^ in comparison to pure Zsw^48^ even without addition of Mg^2+^. Adding Mg^2+^ slightly reduced migration speed, while addition of ligand slightly enhanced migration speed. We attribute the slower migration speed with Mg^2+^ to alteration of ion strength and the slight enhancement upon ligand interaction to a slight compaction of the structure upon pseudoknot stabilization.

### Probing ZMP-binding to transcriptional intermediates of increasing length

**Figure 4.**
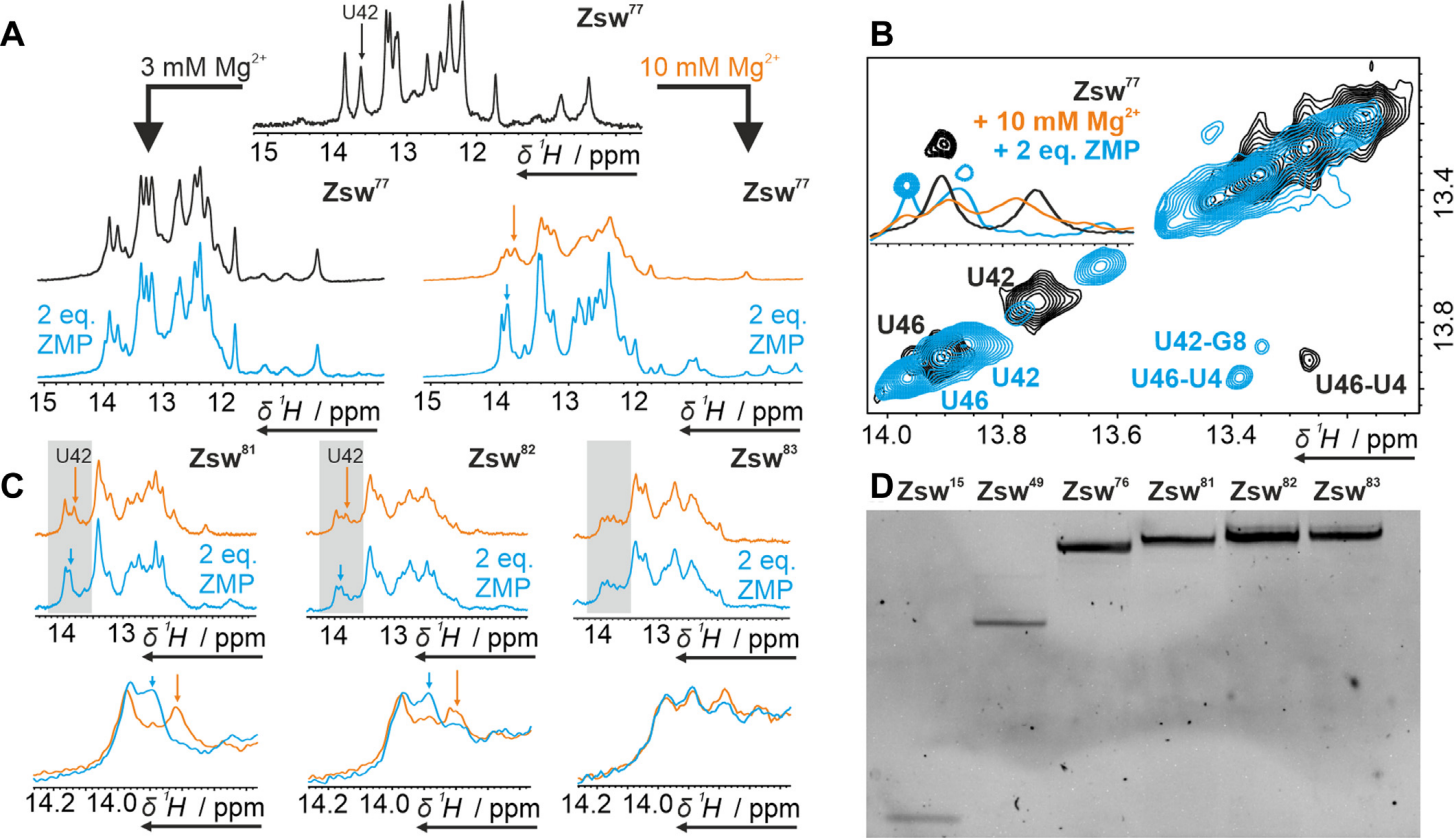
(**A**) ^1^H-1D NMR spectra of Zsw^77^ before and after addition of Mg^2+^ and subsequent addition of 2 eq. ZMP at room temperature. Samples contained 100 μM RNA. (**B**) NOESY and ^1^H-1D data showing an overlay of the Ligand and Mg^2+^ free (black), Mg^2+^ bound (orange, only 1D) and ligand bound (cyan) state of Zsw^77^ at 288K. (**C**) ^1^H-1D NMR spectra of transcripts Zsw^81,82,83^ under influence of 10 mM Mg^2+^ before and after addition of 2 eq. ZMP. U42, which is indicative for binding, is marked with an arrow. ZMP binding is visible for Zsw^81^ and Zsw^82^ while no binding is observed for Zsw^83^. Samples contained 120 μM RNA. Zooms of the regions marked grey in top spectra are displayed in bottom spectra. (**D**) Denaturing PAGE of RNAs Zsw^15^, Zsw^49^, Zsw^76^ and Zsw^81–83^. Gel contained 12 % PAA and 7 M urea. The full gel is shown in Supplementary Figure S8.

We investigated binding of ZMP to potentially critical transcriptional intermediates of the *pfl* riboswitch by NMR spectroscopy. In the crystal structure of the riboswitch (25), a Mg^2+^-ion is detected in close proximity to the binding pocket and ITC studies by Patel *et al.* suggested strong dependence of ZMP affinity on Mg^2+^ concentration (25). Thus, we assumed structural changes to take place upon addition not only of ZMP, but also of Mg^2+^, which were detectable as CSP on imino proton resonances. For two samples of Zsw^77^, we added either 3 mM or 10 mM of MgCl_2_ ([Mg^2+^]:[RNA] = 15/50:1), respectively, before adding two equivalents ([RNA]:[ZMP] = 1:2) of ligand. In both cases, CSPs to the downfield region were observed for all proton signals after addition of Mg^2+^. However, these shifts were accompanied by signal broadening at 10 mM Mg^2+^. After adding two equivalents of ligand, signal line widths decreased and the signals shifted non-uniformly for the 10 mM Mg^2+^ sample. In contrast, the 3 mM Mg^2+^ sample showed no spectral changes upon ZMP addition (Figure 4A). This confirms the observations previously reported (26). The Mg^2+^-induced increase in signal line widths was reversed by the addition of ZMP. The imino proton resonance of U42 represents a potent probe for ligand binding, as it is well separated from the other signals in the spectrum and close enough to the ligand binding pocket to show significant CSPs upon interaction. In the pure RNA sample, it was observed as a strong signal at 13.77 ppm (Figure 4B black spectra). However, upon addition of Mg^2+^, an additional signal appeared at 13.9 ppm with the two populations in an estimated 1:1 ratio (Figure 4B orange spectrum). Addition of ligand than led to loss of the signal at 13.77 ppm and increase of the signal at 13.9 ppm accompanied by a small downfield shift (Figure 4B cyan spectra). These effects might be attributed to the Mg^2+^-dependent pre-formation of a conformationally flexible binding-competent conformational state, which is subsequently stabilized by ligand interaction. When Mg^2+^ was added to Zsw^48^ no second conformation or line broadening was observed (Supplementary Figure S4). For Zsw^101^, no response to ligand or magnesium could be detected (Supplementary Figure S5).

We investigated transcriptional intermediates between the fully binding-competent Zsw^77^ and the binding-incompetent Zsw^101^ RNA for ligand binding. Ligand binding is linked to pseudoknot formation, which competes with terminator stem formation at increasing transcript length. From investigation of the secondary structure we could deduce that the shortest potential construct, to form a closing base-pair of a terminator stem-loop is Zsw^81^, and no additional stabilization for the terminator conformation can be expected beyond Zsw^91^. Accordingly, titration experiments were performed on constructs of lengths between 81 nt and 91 nt, at 10 mM Mg^2+^ and up to 2 eq. ZMP. The constructs were prepared by shifting the reverse primer 3′-end from 5′ to 3′ of the template, achieving different lengths of template DNA for *in vitro* transcription by PCR. *In vitro* transcriptions according to the protocol of Helmling *et al.* (30) allowed quick and homogeneous RNA preparation as shown by denaturing PAGE (Figure 4D and Supplementary Figure S6).

NMR titration (Figure 4C) of Zsw^81^ showed similar CSPs as for Zsw^77^. In the ZMP-free form, under influence of Mg^2+^, a structural equilibrium was observed with U42 signals at 13.77 and 13.9 ppm as in spectra of Zsw^77^. As opposed to the 1:1 ratio observed in Zsw^77^, the binding-competent state was less populated in Zsw^81^, observed from the lower intensity of the U42 signal at 13.9 ppm. Lucks *et al.* showed that the addition of a nucleotide enabling the formation of a one base-pair terminator stem partially destabilizes the antiterminator conformation (28). This effect would explain the spectral alterations observed in the ZMP-free samples of Zsw^77^ and Zsw^81^. However, ligand binding was clearly detectable by chemical shift change of U42. In contrast to the spectrum of ligand-bound Zsw^77^, considerable line-broadening was observed even in the bound state, which suggests population of two or multiple states with interconversion kinetics in the intermediate time regime. For Zsw^82^, the effect of ligand addition was already minor compared to Zsw^81^, observed as ∼50% lower differences in peak intensities. Alongside, we observed increased spectral complexity and line-broadening. Zsw^83^, able to form 3 base-pairs of the terminator hairpin, showed a further shift of the conformational ensemble towards the terminator conformation. Adding ZMP did not lead to any significant structural alteration anymore, which indicates that the terminator conformation was already stable enough to fully outcompete the ligand-binding competent antiterminator conformation. This was also observed for ZMP titration to constructs Zsw^84–90^, which did not show any spectral changes upon ligand addition (Supplementary Figure S7).

**Figure 5.**
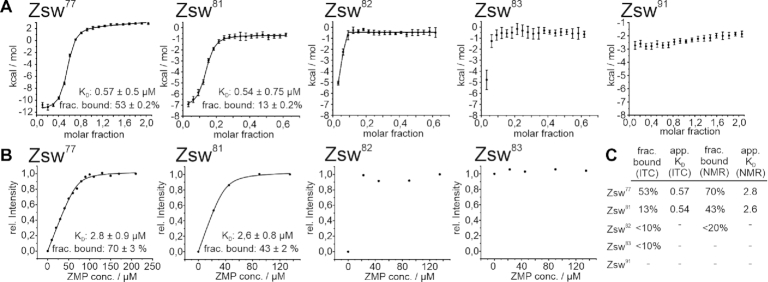
(**A**) ITC titration curves of ZMP to constructs Zsw^77^, Zsw^81–83^ and Zsw^91^. Dissociation constants determined by the fit are annotated as applicable. (**B**) NMR titration curves of ZMP to constructs Zsw^77^, Zsw^81–83^ and Zsw^91^. (**C**) Table summarizing the results of the titration experiments. Values for Zsw^82^ and Zsw^83^ were estimated from the curves displayed. Errors, obtained from the fitting procedures are displayed with the respective curve.

We performed ITC measurements (Figure 5A) on the RNA constructs which showed ligand binding according to NMR titration experiments to determine the equilibrium binding constants. In most cases, riboswitch aptamers exhibit a 1:1 stoichiometry for ligand binding. However, conformational equilibria between binding competent and binding incompetent folds, as observed by NMR, decrease the effective concentration of ligand-binding competent RNA conformations. Accordingly, the data were fitted to accommodate to possible binding incompetent fractions by leaving the stoichiometric parameter variable. Thus, not only the dissociation constants, but also the relative binding competent fractions of the RNA could be obtained. Dissociation constants for Zsw^77^ and Zsw^81^ were 0.57 and 0.54 μM respectively, which is in good agreement with 1 μM obtained by Ferré-d’Amaré *et al.* for the *Fusobacterium ulcerans* ZMP-sensing riboswitch (26). Dissociation constants obtained from NMR measurements (Figure 5B) were slightly higher with 2.78 μM for Zsw^77^ and 2.58 μM for Zsw^81^. While the affinity remains constant throughout the two different RNA constructs, the amount of binding competent RNA largely differs. By ITC, the binding-competent fractions are 53 % (Zsw^77^) and 13% (Zsw^81^), while in NMR measurements, a decrease from 70% to 43% was observed (Figure 5C). However, the degree of decreased binding differs largely between the two methods ITC and NMR-spectroscopy with a relative decrease of roughly 75% in ITC and only 40% in NMR measurements from Zsw^77^ to Zsw^81^. A possible explanation could be the slow time-scale of equilibration between measurements. While ZMP was added with few minutes of waiting time between injections in ITC, NMR experiments take around a factor of 10 longer. For Zsw^82^, only a very minor binding competent population was observable both in ITC and NMR titrations, which prevented curve fitting and thus the determination of a dissociation constant or the exact population of states. However, we estimated from curve shapes of constructs longer than 81 nt that the amount of binding competent RNA must be below 10 % in ITC measurements, up to Zsw^91^, which showed no interaction at all.

## DISCUSSION

In this study, we explored the folding landscape of the ZMP-sensing riboswitch from *T. carboxydivorans* by NMR spectroscopy and ligand binding affinities by ITC. From ^1^H,^1^H-NOESY experiments imino proton assignment of Zsw^48^ could be achieved and transferred to the longer construct Zsw^77^, enabling the observation of ligand binding on the well separated resonance of U42. We additionally could confirm pre-formation of the pseudoknot and binding competence of an *in-trans* construct in which the linker between P1 and P3 was deleted. We observed only two G-residues of the possible four G–C base-pairs in the pseudoknot, suggesting a lower stability at one edge of the pseudoknot helix. Taking the crystal structure (25) into account, it is highly likely that G16–C62 and G17–C61 are less stable than G14–C65 and G15–C64 base-pairs, since G14 sits directly adjacent to the highly structured binding-pocket, while G17–C61 constitutes the closing base-pair of the pseudoknot helix. As reported previously (25), binding was only achieved at a high Mg^2+^ concentration of 10 mM, where substantial line broadening of NMR signals was observed, while the initial linewidth was regained after ZMP addition. Upon addition of 10 mM Mg^2+^, we additionally observed a structural equilibrium between two states, in which one state showed reporter peak shifts similar to the ligand-bound and the other one similar to the free state. Upon ligand interaction the system collapsed into the ligand bound state with minimal chemical shift alteration compared to the Mg^2+^-bound state. We therefore propose an additional Mg^2+^-dependent pre-formation of the binding pocket, which goes beyond just pseudoknot-formation, since pseudoknot formation is already observed without Mg^2+^ addition. However, pseudoknot formation is required for this additional Mg^2+^-stabilized state, since Zsw^48^ does not show any spectral alteration upon Mg^2+^ addition, in the absence of Zsw^15^. The Mg^2+^ binding step can thus be considered an intermediate step, taking place after pseudoknot formation and facilitating ligand interaction and therefore final stabilization of the binding pocket.

Longer constructs showed only slight alteration in the population of these states in the presence of Mg^2+^. However, the amount of RNA which refolded to the ligand-bound state upon ZMP addition plummeted between Zsw^81^ and Zsw^82^, visible from quantitative NMR and ITC data. This further indicated that the existence of a Mg^2+^-induced pre-bound state is decoupled from the pseudoknot-terminator equilibrium, but both must be present to allow ligand binding. Lucks *et al.* reported that already the addition of a single nucleotide, competing with P3 helix stability (i.e. Zsw^81^), leads to partial ZMP binding incompetence, which we also observe via ITC and NMR spectroscopy. However, for this construct, absolute values differed largely between ITC and much slower NMR measurements. We therefore suspect very slow folding kinetics on a timescale of minutes between a binding incompetent pre-terminator state back to a binding competent state if binding competent RNA is removed from the equilibrium by ligand interaction. This would render the pre-terminator state kinetically trapped in a biological context. Consequently, it is expected that RNA displaying formation of a single terminator base-pair could already be predominantly binding incompetent *in vivo*, where fast progress of transcription limits the refolding time available. In comparison, Zsw^82^ displays strongly reduced binding competence and an almost full population of the terminator state, leaving Zsw^81^ as the only construct with considerable population of both states. Therefore, in a biological context, the transition to the binding incompetent state is expected to be comparably sharp in contrast to other examples like the deoxyguanosine-sensing riboswitch, which displays a smoother transition (19). The high stability of short pre-terminator constructs leads to a very short window for ligand interaction during transcription of the riboswitch, which is only possible while RNA of lengths 74–76 to 81 nt is accessible to refolding and therefor ligand interaction. Linker length between P3 and terminator stem could therefore be an important feature that tunes riboswitch activity over genetic occurrences as in other prokaryotes, where this length strongly varies (25). It should be noted, that in terms of co-transcriptional folding, 14 nucleotides, starting from the active side are involved in the transcription elongation complex (TEC), with 9 nt hybridized to DNA and 5 nt located in the exit channel (38). In principle 2 to 3 more residues could be involved in RNA folding and removed from the DNA:RNA duplex without the TEC dissociating (39), leaving 7 to 8 residues in the exit channel with potential for interaction. While these residues are unable to interact in tertiary interactions with further upstream residues, as in the pseudoknot structure, RNA secondary structure formation inside the exit channel is observed in some SHAPE-seq (40) and smFRET (41) experiments for specific constructs. The assessment of the ability of the Zsw terminator structure to form in the exit channel of the *T. carboxydivorans* RNAP is beyond the scope of this project. Therefore, nucleotide numbers mentioned here, refer to residues accessible to folding in a manner comparable to *in vitro* folding, so most likely outside the RNAP exit channel. Consequently, during folding of the first pre-terminator base-pair at position 81, the RNAP active site will reside at position 91–93, the position of the terminator poly-U stretch. Therefore, transcriptional speed might be additionally modulated by transcriptional pausing, a well studied effect in transcriptional regulation and action of rho-independent terminators (18,42).

**Figure 6.**
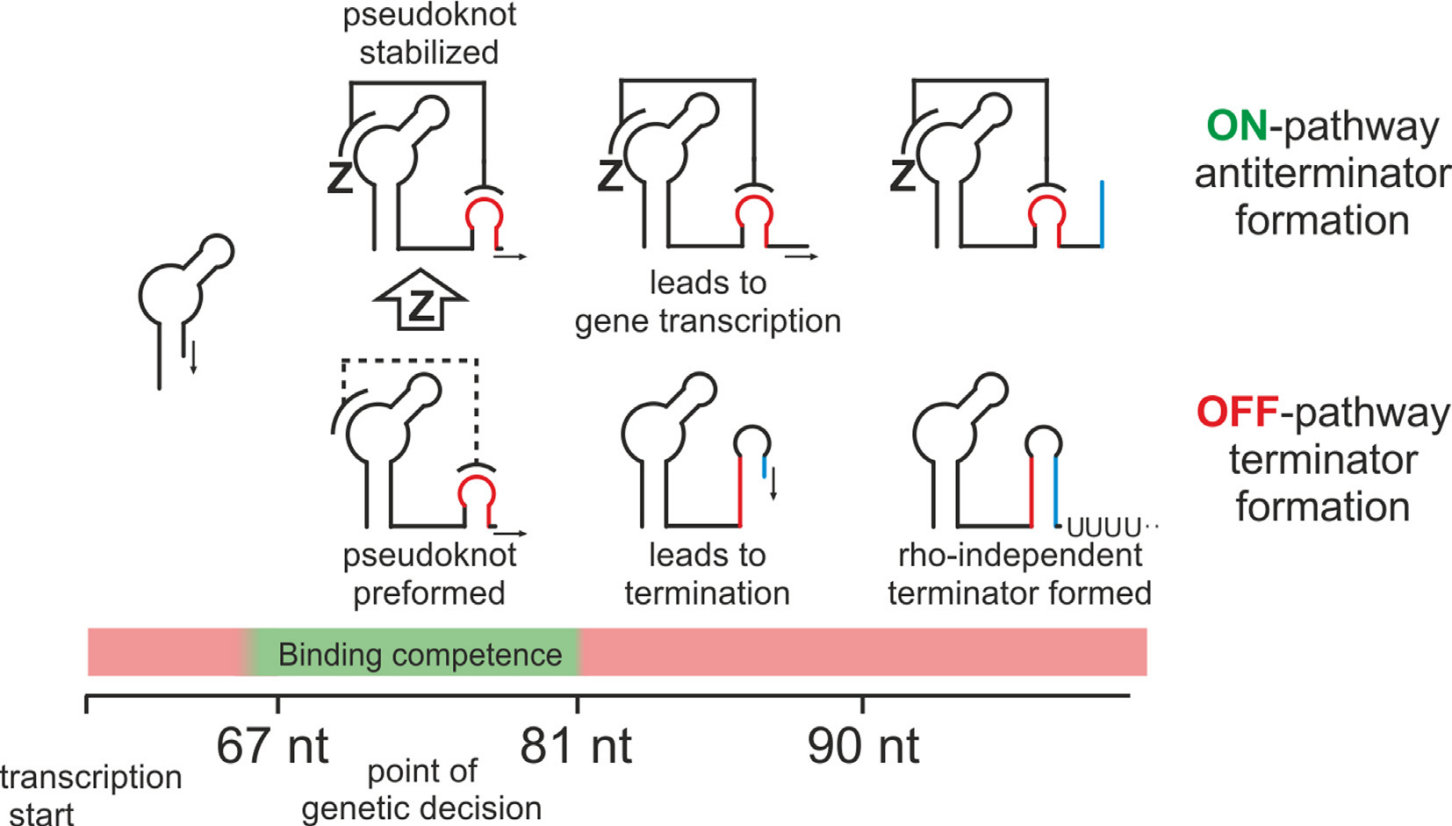
Schematic representation of the model for genetic control employed by the ZMP-sensing riboswitch from *T. carboxydivorans*. The point of genetic decision during transcription of nucleotides 67–81 is characterized by binding competence. At this stage, the pseudoknot is preformed. Binding of the cognate ligand ZMP leads to stabilization of the pseudoknot bearing antiterminator structure and subsequent gene transcription. No binding leads to formation of the terminator structure and rho-independent transcription termination.

We propose a model (Figure 6) in which a pre-formed pseudoknot is further stabilized by ligand interaction, leading to anti-termination of the riboswitch, which is in agreement with the results Lucks *et al.* obtained using SHAPE-seq (28). The time-window of the binding competent conformation is, due to the very stable pre-terminator Zsw^82^, comparably short, featuring structural equilibria only at a transcript length of 81 nt. This implies that for biological function to be carried out correctly, the binding event must be considerably fast compared to transcript elongation (possibly modulated by trans acting factors such as Nus proteins), which could be an interesting premise for further kinetic experiments. With the terminator fold being thermodynamically more stable than the ligand-bound antiterminator fold, the stabilization of the pseudoknot established by ZMP addition can be considered temporary and therefore kinetically driven. This type of regulation is a common motif observed in riboswitches such as fluoride- (43), FMN- (44), deoxyguanosine- (19) or guanosine-sensing (45) riboswitches. Biological function of the riboswitch is therefore expected to be heavily tuned by terminator folding kinetics with and without the influence of ZMP.

To resolve these kinetics, real-time NMR measurements can be performed by rapid addition of interactors or by introduction and subsequent elimination of photolabile protecting groups. However, these studies require sufficient exploration of the underlying system in equilibrium state. We herein provided the NMR spectroscopic basis for such studies, showing important key players of the conformational landscape of the *pfl* riboswitch from *T. carboxydivorans* and their structural characteristics at atomic resolution.

## Supplementary Material

gkaa427_Supplemental_File
