# Supplementary material for: The conformational landscape of transcription intermediates involved in the regulation of the ZMP-sensing riboswitch from Thermosinus carboxydivorans
Source: Nucleic Acids Res. 2020 Jun 1;48(12):6970–9. doi: 10.1093/nar/gkaa427 (PMC7337938; doi:10.1093/nar/gkaa427)
Supplement: gkaa427_Supplemental_File [file gkaa427_supplemental_file.docx]

Supplementary data

**The conformational landscape of the transcription intermediates of the ZMP-sensing riboswitch from *Thermosinus carboxydivorans***

Oliver Binas,^[a]^ Tatjana Schamber^[a]^ and Harald Schwalbe*^[a]^

[a] O. Binas T. Schamber,, Prof. Dr. H. Schwalbe
Institute for Organic Chemistry and Chemical Biology
Goethe University Frankfurt
Max-von-Laue Straße 7, 60438 Frankfurt
E-mail: schwalbe@em.uni-frankfurt.de

Table of Contents:

- **Supplemmentary Table S1**. Summary of all forward and reverse DNA primers used in this study.
- **Supplementary Figure S1**. Imino regions of ^1^H,^1^H-NOESY spectra of Zsw^48^ and Zsw^77^.
- **Supplementary Figure S2.** ^15^N-edited and conventional ^1^H-1D NMR spectra of ^15^N-Zsw^48^+^14^N-Zsw^15^ and conventional ^1^H-1D of Zsw^15^ in the presence of 10 mM Mg^2+^.
- **Supplementary Figure S3.** 12 % native PAGE of Zsw^48^ in the presence of 1eq Zsw^15^, 10 mM Mg^2+^ and 1 eq. ZMP.
- **Supplementary Figure S4.** ^1^H-1D spectra of Zsw^48^ in presence and absence of 10 mM Mg^2+^ and 1 eq Zsw^15^.
- **Supplementary Figure S5.** ^1^H-1D spectra of Zsw^101^ in presence and absence of 1 eq ZMP.
- **Supplementary Figure S6.** 12 % native PAGE of Zsw^81-91^.
- **Supplementary Figure S7.** 1H-1D spectra of Zsw^81-91^ in presence and absence of 1 or 2 eq. ZMP.
- **Supplementary Figure S8.** 12% denaturing PAGE of Zsw^15^, Zsw^48^, Zsw^77^ and Zsw^81-83^.

**Supllementary Table S1**. Summary of all forward and reverse DNA primers used in this study, as well as the RNA sequence of the full length ZMP riboswitch (Zsw^100^ RNA).

| Construct | Forward Primer | Reverse Primer |
| --- | --- | --- |
| Zsw^48^ | 5’-TAATACGACTCACTATAGG-3’ | 5’-CGGATACAGTCCACGTGGTTGCTTC-3’ |
| Zsw^77^ |  | 5’-TTTTTGCCCAGGCGGTCGGCTTTTC-3’ |
| Zsw^81^ |  | 5’- [CT]AATTTTTGCCCAGGCGGTCGGC-3’ |
| Zsw^82^ |  | 5’- [GC]TAATTTTTGCCCAGGCGGTCGG-3’ |
| Zsw^83^ |  | 5’- [GG]CTAATTTTTGCCCAGGCGGTCG-3’ |
| Zsw^84^ |  | 5’- [GG]GCTAATTTTTGCCCAGGCGGTC-3’ |
| Zsw^85^ |  | 5’- [TG]GGCTAATTTTTGCCCAGGCGGT-3’ |
| Zsw^86^ |  | 5’- [CT]GGGCTAATTTTTGCCCAGGCGG-3’ |
| Zsw^87^ |  | 5’- [CC]TGGGCTAATTTTTGCCCAGGCG-3’ |
| Zsw^89^ |  | 5’- [CG]CCTGGGCTAATTTTTGCCCAGG-3’ |
| Zsw^90^ |  | 5’- [CC]GCCTGGGCTAATTTTTGCCCAG-3’ |
| Zsw^91^ |  | 5’- [AC]CGCCTGGGCTAATTTTTGCCCA-3’ |
| Zsw^101^ | 5’-TAATACGACTCACTATAGGATACA GGACTGGCG-3’ | 5’-AAAAATAAATACCGCCTGGGCTAATTT TTGCCC-3’ |
| Zsw^100^ (RNA) | 5’‑GGAUACAGGACUGGCGGAUUAGUGGAAGCAACCACGUGGACUGUAUCCGAAGAAAAGCCGACCGCCUGGGCAAAAAUUAGCCCAGGCGGUAUUUAUUUUU-3’ | |


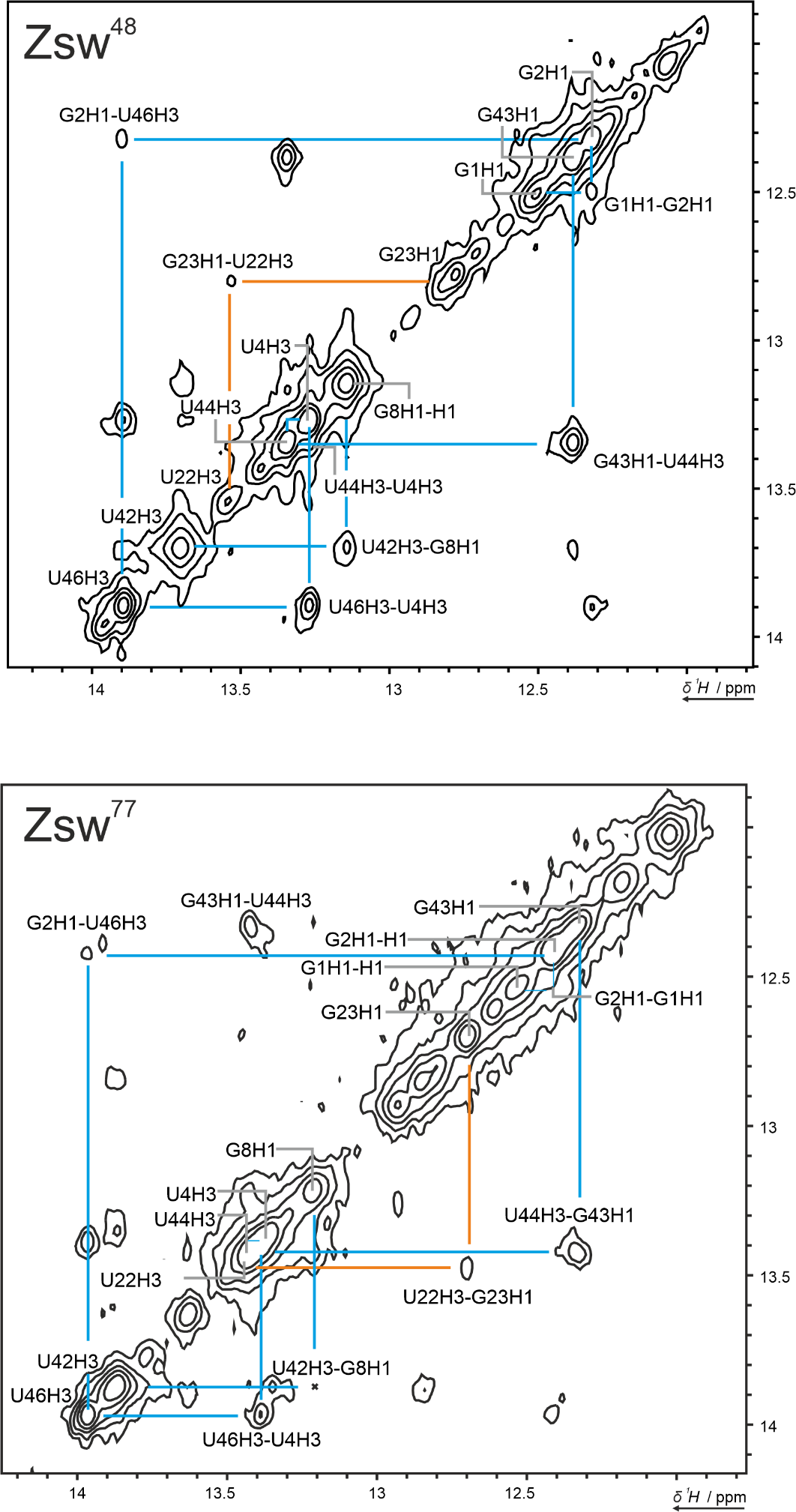


**Supplementary Figure S1.** Imino regions of ^1^H,^1^H-NOESY spectra of Zsw^48^ (a) and Zsw^77^ (b) annotated with assignment. Color-coding indicates resonances from either P1 (cyan) or P2 (orange). Data were measured at 600 MHz, 2048 x 640 points and 184 scans and 800 MHz, 2048 x 512 and 256 points for Zsw^48^ and Zsw^77^ respectively. Samples contained 800 µM (Zsw^48^) or 400 µM (Zsw^77^) RNA, 25 mM potassium phosphate buffer (pH 6.2), 50 mM KCl and 10 % D_2_O.


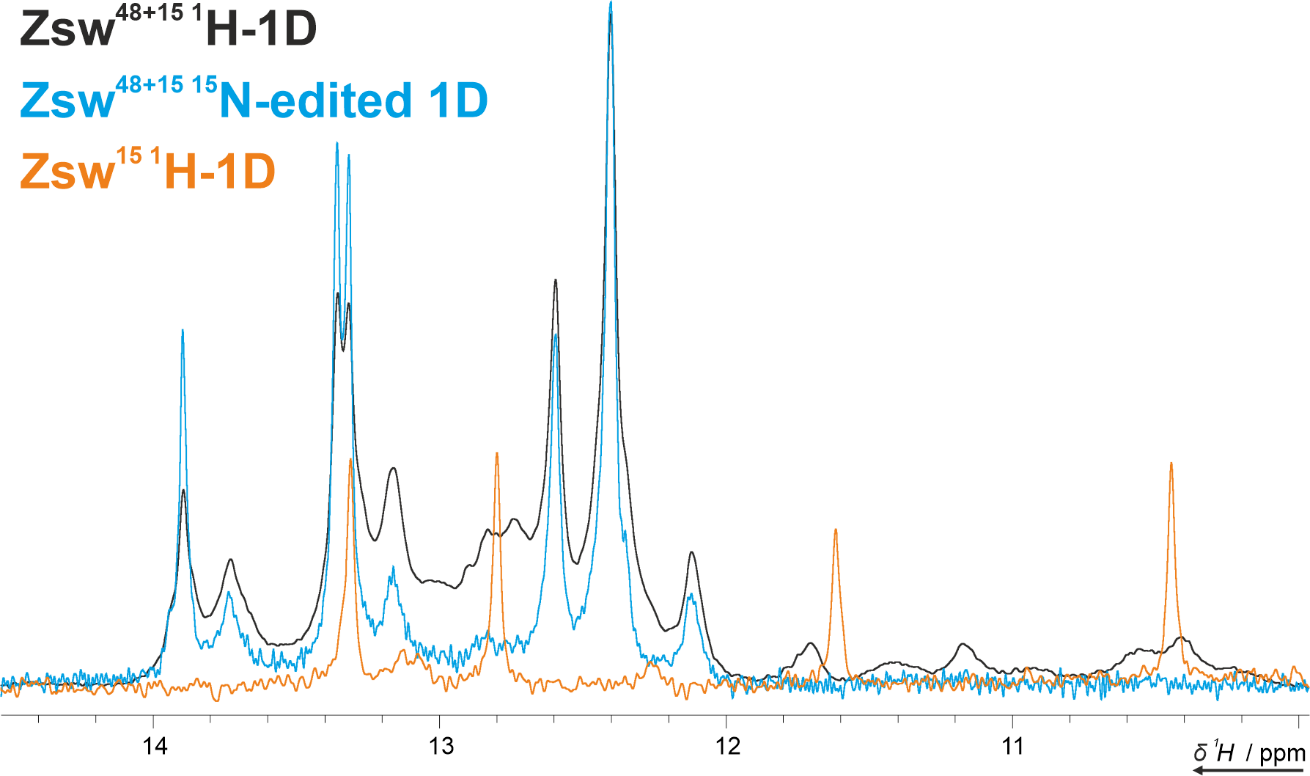


**Supplementary Figure S2.** ^15^N-edited and conventional ^1^H-1D NMR spectra of ^15^N-Zsw^48^+^14^N-Zsw^15^ and conventional ^1^H-1D of Zsw^15^ in the presence of 10 mM Mg^2+^. Samples contained 100 µM RNA, 25 mM potassium phosphate buffer (pH 6.2), 50 mM KCl.


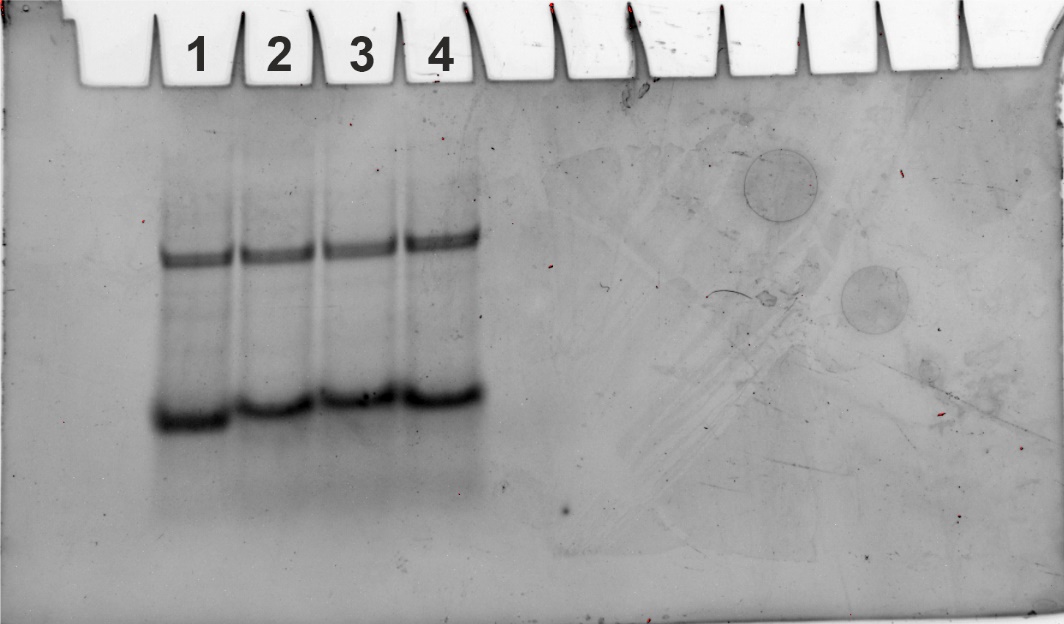


**Supplementary Figure S3.** 12 % native PAGE of Zsw^48^ in the presence of 1eq Zsw^15^, 10 mM Mg^2+^ and 1 eq. ZMP. Pockets were loaded with 200 nmol Zsw^48^ (lane 1) and additionally 1 eq. Zsw^15^ (lane 2), 10 mM Mg^2+^ (lane 3) and 1 eq. ZMP (lane 4). Upper bands are residual double stranded DNA from the preparation of Zsw^48^.


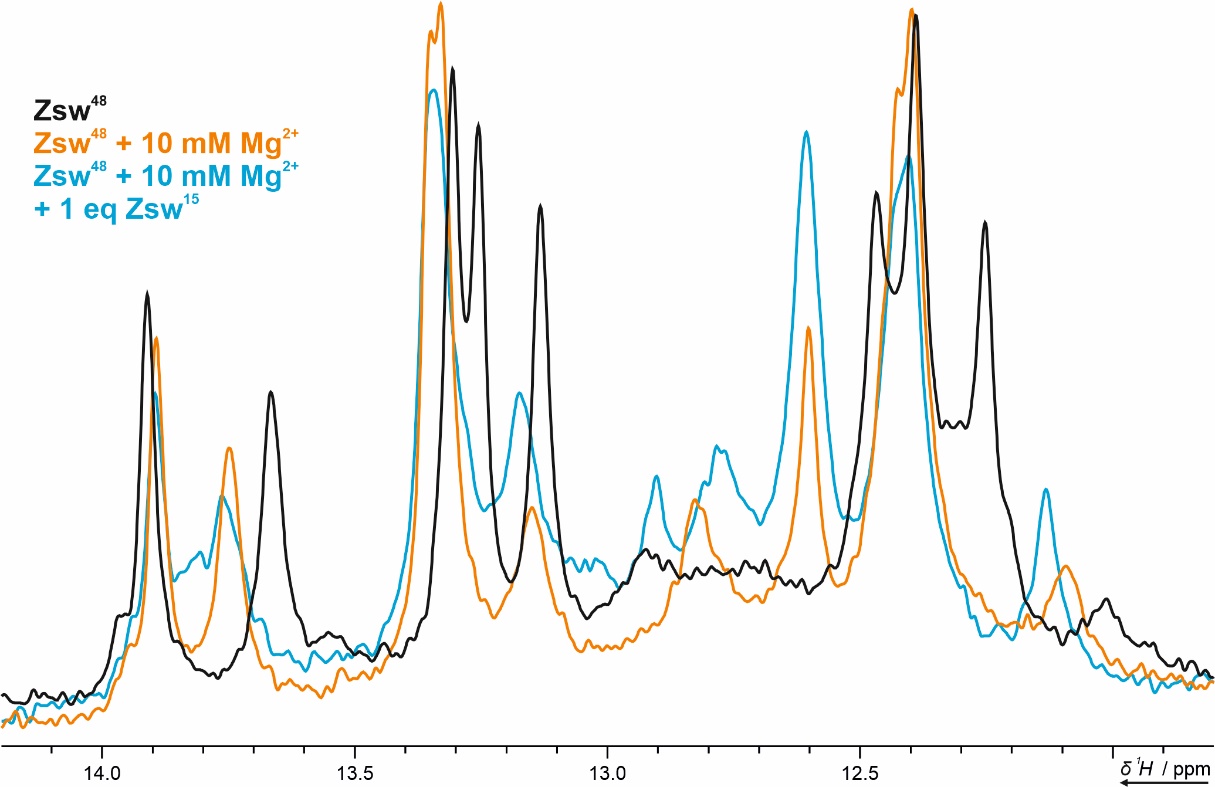


**Supplementary Figure S4.** ^1^H-1D spectra of Zsw^48^ in presence and absence of 10 mM Mg^2+^ and 1 eq Zsw^15^. Samples contained 450 µM RNA, 25 mM potassium phosphate buffer (pH 6.2), 50 mM KCl 10 mM Mg^2+^ and 10% D2O.


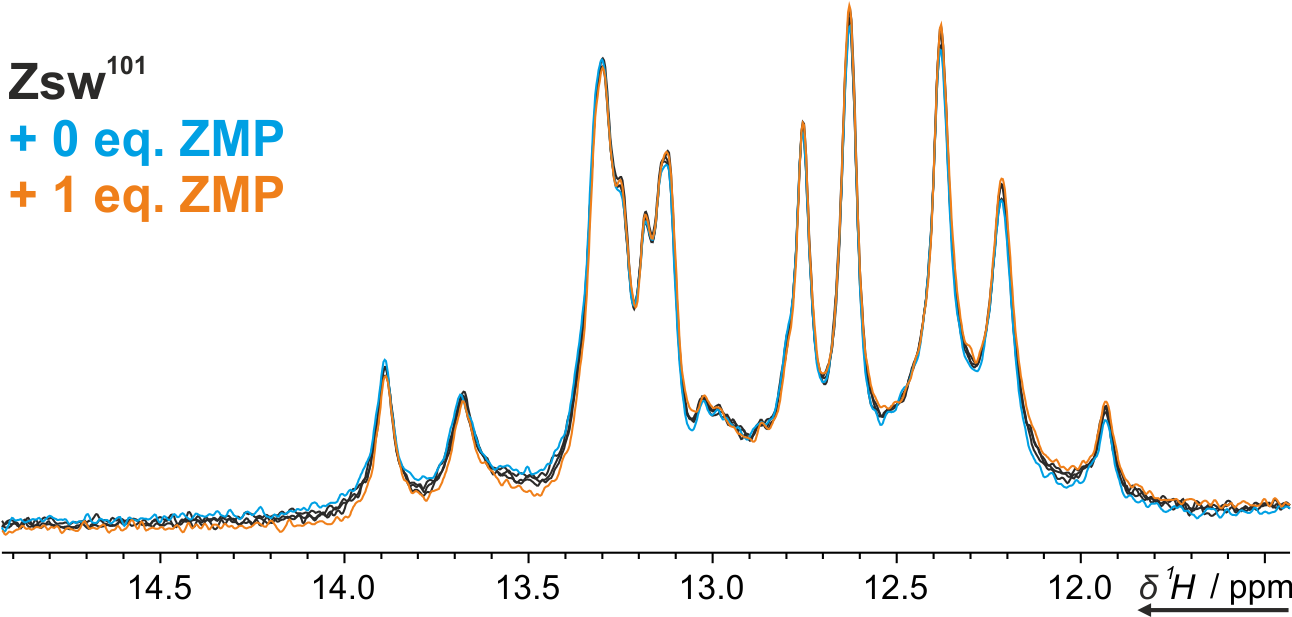


**Supplementary Figure S5.** ^1^H-1D spectra of Zsw^101^ in presence and absence of 1 eq ZMP. Samples contained 150 µM RNA, 25 mM potassium phosphate buffer (pH 6.2), 50 mM KCl 3 mM Mg^2+^ and 10% D2O.


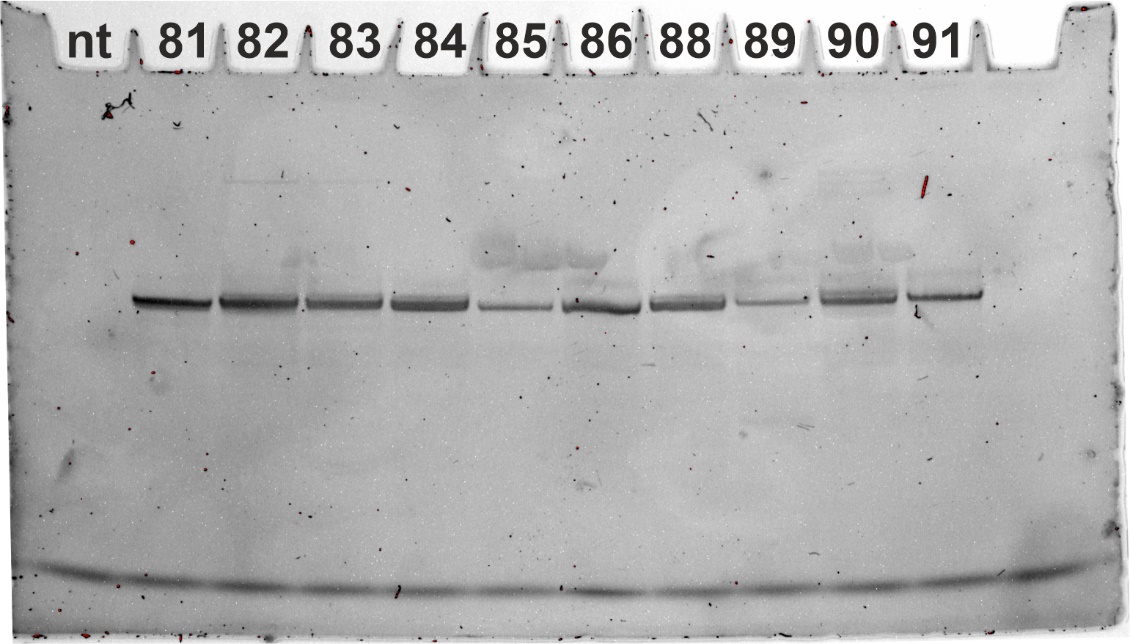


**Supplementary Figure S6.** 12 % native PAGE of Zsw^81-91^. Pockets were loaded with 0.15 µl RNA transcription mix.


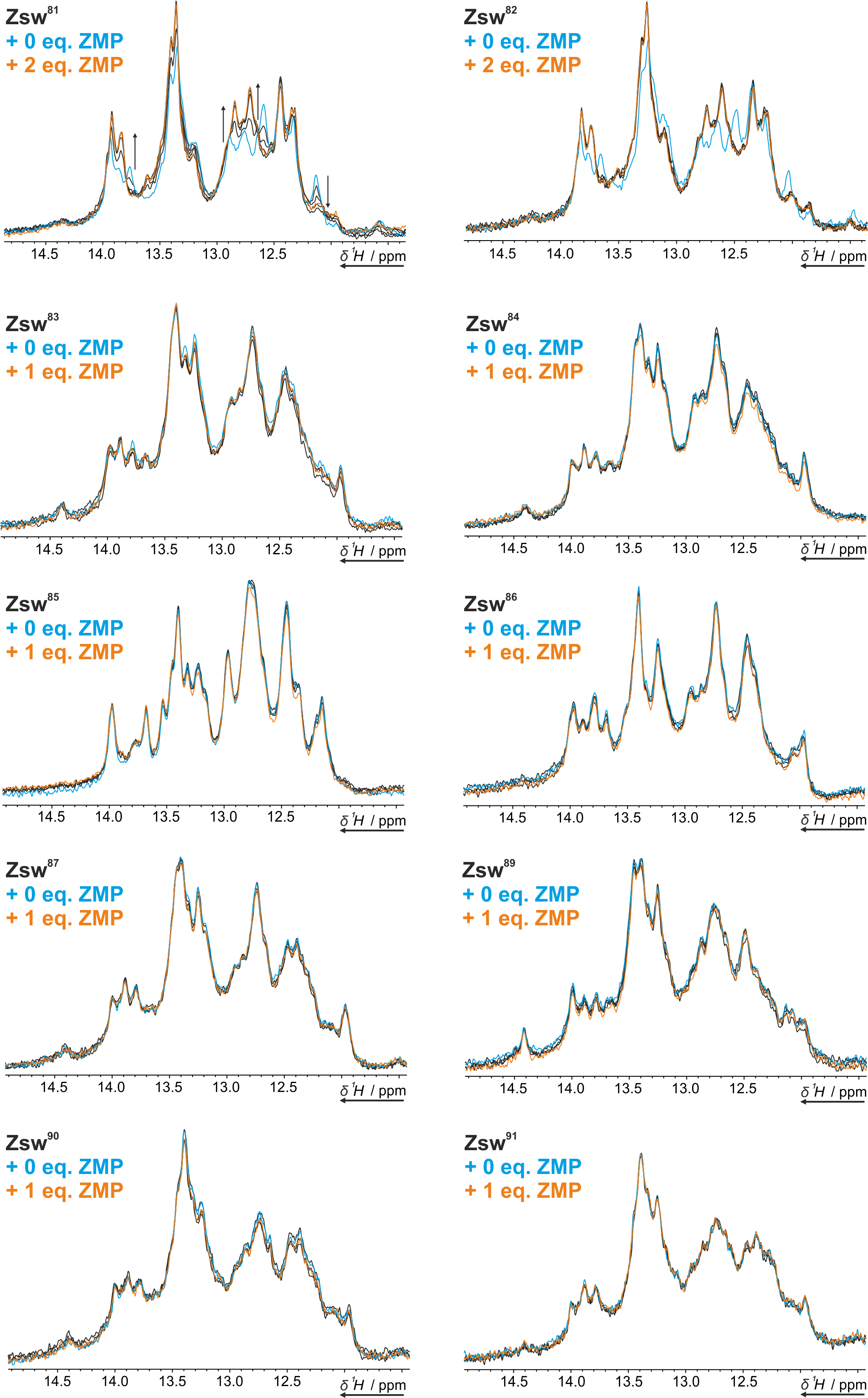


**Supplementary Figure S7.** 1H-1D spectra of Zsw^81-91^ in presence and absence of 1 or 2 eq. ZMP. Binding is observed only for Zsw^81^ and Zsw^82^. Samples contained 100-120 µM RNA, 25 mM potassium phosphate buffer (pH 6.2), 50 mM KCl 10 mM Mg^2+^ and 10% D2O.


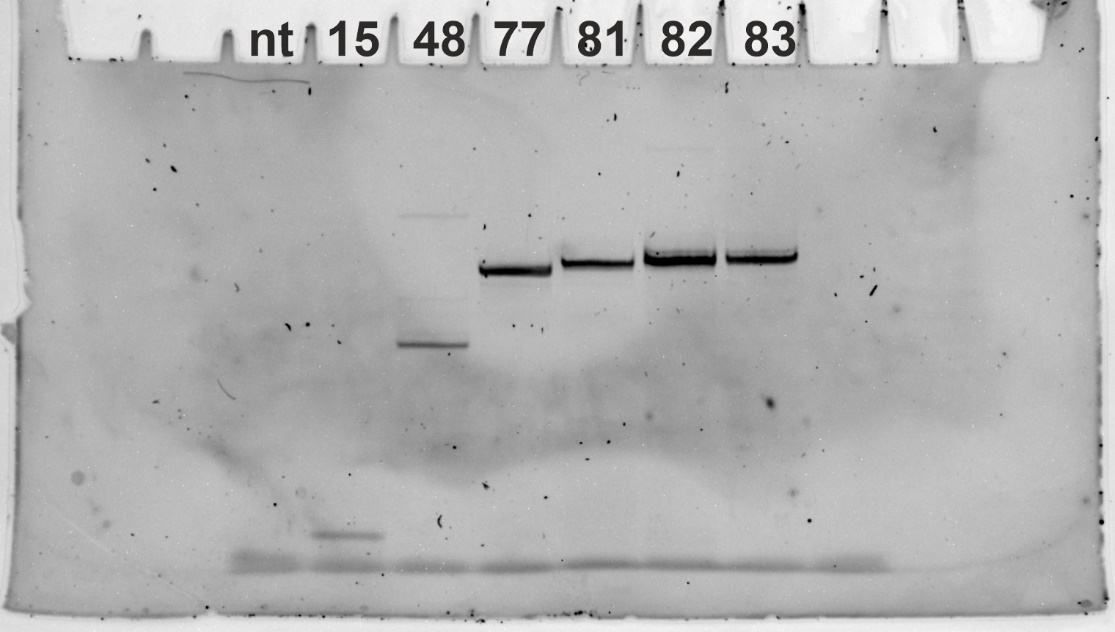


**Supplementary Figure S8.** 12% denaturing PAGE of Zsw^15^, Zsw^48^, Zsw^77^ and Zsw^81-83^. Pockets were loaded with approx. 200 nmol of the respective RNA. Smaller constructs are naturally stained less by the visualization agent and therefore appear weaker.
